# Within-Region Versus Out-of-Region Cancer Care: Healthcare Patterns and Clinical Outcomes in South Korea

**DOI:** 10.3390/cancers18172879

**Published:** 2026-09-06

**Authors:** Woo-Ri Lee, Jinsoo Shin, Junghwa Kim, Kyu-Tae Han

**Affiliations:** Division of Cancer Control and Policy, National Cancer Control Institute, National Cancer Center, Goyang 10408, Republic of Korea; wind0747@gmail.com (W.-R.L.); 77717@ncc.re.kr (J.S.); jjhh11@ncc.re.kr (J.K.)

**Keywords:** cancer care centralization, out-of-region care, healthcare equity, health utilization, cancer outcomes

## Abstract

**Background/Objectives:** Patient concentration in high-volume tertiary hospitals in the capital region of South Korea has become a major health policy concern. We examined the associations of out-of-region cancer care with healthcare utilization, healthcare expenditure, and clinical outcomes. **Methods:** We included 303,443 patients newly diagnosed with one of six major cancers between 2012 and 2020 using the Cancer Public Library Database of the Korea Clinical Data Utilization Network for Research Excellence project. Out-of-region cancer care was defined as initial treatment received outside the patient’s residential region. Multivariable regression models evaluated healthcare utilization, healthcare expenditure, and clinical outcomes. Sensitivity analyses considered pretreatment care-seeking fragmentation, and subgroup analyses were performed by cancer stage. **Results:** Out-of-region cancer care was associated with higher healthcare expenditure (relative risk [RR], 1.02; 95% confidence interval [CI], 1.02–1.03), delayed treatment initiation (odds ratio [OR], 1.19; 95% CI, 1.15–1.22), and lower 1-year (hazard ratio [HR], 0.88; 95% CI, 0.84–0.92) and 5-year (HR, 0.93; 95% CI, 0.91–0.96) all-cause mortality. A modest association with increased emergency department visits was observed in the main analysis (HR, 1.03; 95% CI, 1.01–1.07), although this association was not robust in the sensitivity analyses. These associations with healthcare utilization were stronger among patients with pretreatment care-seeking fragmentation and those with localized or regional cancer. **Conclusions:** Out-of-region cancer care was associated with lower mortality but higher healthcare utilization. Strengthening regional cancer care capacity and coordinated referral pathways may improve continuity of care while ensuring equitable access to specialized cancer services.

## Simple Summary

Many patients with cancer receive out-of-region cancer care to access specialized cancer services, particularly at high-volume tertiary hospitals. However, this pattern may involve both benefits and burdens. In this nationwide study using linked cancer registry and health insurance claims data, out-of-region cancer care was associated with lower all-cause mortality but higher healthcare utilization, including greater healthcare expenditure, delayed treatment initiation, and more emergency room visits. These adverse associations were more pronounced among patients who experienced pretreatment care-seeking fragmentation, suggesting that reduced continuity of care before treatment may contribute to less efficient cancer care. Our findings indicate that cancer care policies should strengthen regional cancer care capacity and improve coordinated referral systems to reduce unnecessary fragmentation while maintaining access to specialized cancer services and promoting regional equity.

## 1. Introduction

Cancer is one of the most common diseases and the leading cause of death in South Korea, with 522.9 newly diagnosed cases per 100,000 population in 2023 and 294.6 deaths per 100,000 population in 2024 [1]. Since 2004, the Korean government has sought to strengthen the regional cancer care capacity by establishing regional cancer centers [2]. In parallel, the expansion of National Health Insurance (NHI) coverage between 2005 and 2009 substantially reduced financial barriers to cancer care by lowering the copayment rate for cancer treatment from 20% to 5% [3]. However, despite these policy efforts, regional disparities in cancer care have persisted. The uneven distribution of the medical infrastructure and patient concentration in large hospitals located in the capital region have become major health policy concerns in South Korea [4,5].

Cancer care has become a major strategic service for hospitals because it involves high-cost technologies, specialized multidisciplinary services, and substantial institutional investments. Leading tertiary hospitals in Seoul have expanded specialized cancer centers and invested heavily in advanced technologies, multidisciplinary care, and specialized services. In particular, several major tertiary hospitals in Seoul, which are often referred to as the Big 5 hospitals, have emerged as dominant providers of cancer care, thus reinforcing the patient concentration in the capital region. This pattern may be understood as a type of competition between hospitals that involves the expansion of high-cost specialized services and infrastructure [6,7].

Consequently, it has become increasingly common for patients diagnosed with severe diseases such as cancer to seek care outside their residential regions, particularly at high-volume tertiary hospitals located in Seoul. According to NHI statistics, 78.5% of patients with newly diagnosed cancer received care in the capital region in 2023; however, only 48.9% of these patients resided in the capital region [8]. Additionally, the outflow of local patients to the Big 5 hospitals has been repeatedly highlighted as a major health policy concern during parliamentary audits.

Although several policies aimed at regionalizing healthcare delivery systems, including cancer care, have been introduced, the government continues to encounter substantial challenges related to population aging and regional depopulation in rural areas. In February 2026, the Ministry of Health and Welfare announced the 5th Comprehensive Cancer Control Plan (2026–2030), which emphasized alleviating the concentration of patients with cancer in the capital region and improving the regional relevance index for treatment across major cancers [9].

Furthermore, according to the Survey on the Perception of Regional Healthcare conducted in 2025, more than 80% of respondents perceived disparities between the capital and noncapital regions as severe and believed that these disparities should be addressed. Previous studies have suggested that patient outmigration and centralization of specialized cancer care may increase travel burden, treatment delays, and socioeconomic costs [10,11,12,13]. However, limited empirical evidence exists regarding whether out-of-region cancer care is associated with differences in healthcare utilization, medical expenditures, and clinical outcomes in South Korea. Therefore, this study investigated patterns of out-of-region cancer care, defined by comparing the location of initial cancer treatment with the patient’s residential region at cancer diagnosis, and examined their associations with healthcare utilization, expenditures, and clinical outcomes among patients with major cancers.

## 2. Materials and Methods

### 2.1. Data and Participants

**Figure 1:**
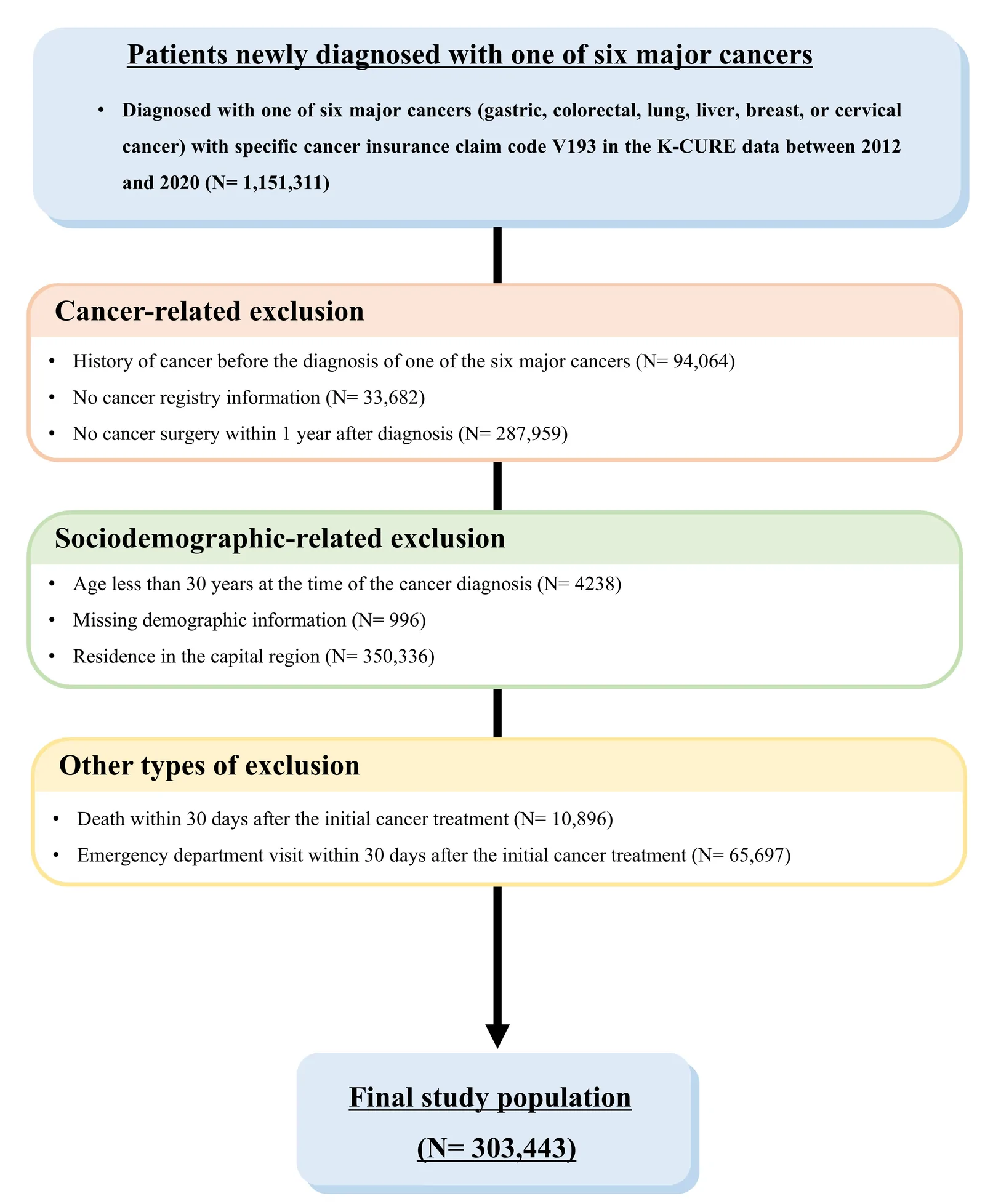
Data flowchart for this study.

This nationwide retrospective cohort study used the Cancer Public Library Database (CPLD) from the Korea Clinical Data Utilization Network for Research Excellence project (K-CURE). The CPLD contains linked nationwide data regarding cancer registration and death, demographics, medical claims, general health examinations, and national cancer screening for approximately 2 million patients with cancer [14]. To investigate out-of-region cancer care patterns and their associations with healthcare utilization and clinical outcomes, we included patients diagnosed with one of six major cancers (gastric, colorectal, lung, liver, breast, or cervical cancer) targeted by the National Cancer Control Program (N = 1,151,311). To identify newly diagnosed cancer cases, we included patients who were newly registered with the cancer-specific copayment reduction code (V193) in the NHI Service claims data between 2012 and 2020. We excluded patients with a history of cancer before the diagnosis of one of six major cancers (N = 94,064), those without cancer registry information (N = 33,682), those who did not undergo surgical cancer treatment within 1 year after diagnosis (N = 287,959), those with missing demographic information (N = 996), those younger than 30 years (N = 4238), those who died or visited the emergency room within 30 days after the initial cancer treatment (N = 76,593), and those residing in the capital region (Seoul, Gyeonggi, or Incheon; N = 350,336). The study population was restricted to patients who underwent surgical cancer treatment within 1 year after diagnosis to establish a more clinically comparable cohort and reduce heterogeneity arising from differences in treatment pathways. For those younger than 30 years, the exclusion was based on the K-CURE age classification, which groups individuals aged 0–29 years into a single category encompassing children, adolescents, and young adults with different cancer types and treatment pathways. A 30-day landmark approach was applied after initial cancer treatment to minimize the influence of acute peri-treatment events, including postoperative complications and other immediate treatment-related events, and to focus on subsequent healthcare utilization and longer-term clinical outcomes. The final study population comprised 303,443 patients (Figure 1).

### 2.2. Variables

#### 2.2.1. Outcome

The primary outcomes of this study were healthcare utilization, medical expenditures, and clinical outcomes. Total medical expenditures, delayed cancer treatment, emergency room visits, and mortality were assessed. Total medical expenditures were defined as cumulative expenditures for outpatient visits and inpatient admissions related to cancer care within 1 year after diagnosis. To account for inflation, all expenditures were adjusted using the annual conversion index and expressed in 2023 values to correspond to the final year of the study data. Delayed cancer treatment was defined as no cancer treatment, including surgery, chemotherapy, and radiotherapy, within 30 days after diagnosis. Emergency room visits were assessed as the time to the first emergency room visit within 1 year after the initiation of the initial cancer treatment. Mortality was defined as all-cause death within 1 year and 5 years after the initial cancer treatment.

#### 2.2.2. Independent Variable

The primary exposure variable was the cancer care utilization pattern, which was categorized as within-region or out-of-region cancer care based on whether patients received cancer treatment inside or outside their residential region. The Residential region was defined according to the administrative district at the time of the diagnosis using 17 provincial-level regions, including metropolitan cities and provinces (Si-Do), in the CPLD. Out-of-region cancer care was defined as receiving initial cancer treatment, including surgery, chemotherapy, and radiotherapy, in an administrative region other than the patient’s residential region based on the location of the treating hospital. Patients who received their initial cancer treatment within their residential region were categorized as receiving within-region cancer care, and those treated outside their residential region were classified as receiving out-of-region cancer care.

We performed a sensitivity analysis to examine whether associations between cancer care utilization patterns and study outcomes differed according to pretreatment care-seeking fragmentation, which was defined as visiting two or more hospitals for cancer-related care between the cancer diagnosis and initiation of the initial cancer treatment. Multi-institution care-seeking during this period was used as a proxy for pretreatment care-seeking fragmentation and may include additional consultations, appropriate referrals, or second-opinion visits before initial treatment was initiated.

To jointly consider treatment location and fragmented care-seeking before treatment, we reclassified patients into the following four mutually exclusive groups: within-region care without pretreatment care-seeking fragmentation (WR/NF); out-of-region care without pretreatment care fragmentation (OR/NF); within-region care with pretreatment care-seeking fragmentation (WR/WF); and out-of-region care with pretreatment care-seeking fragmentation (OR/WF). Then, we repeated multivariable regression analyses using the four-category exposure variable with the WR/NF group as the reference.

#### 2.2.3. Covariates

Covariates included socioeconomic, health-related, and cancer-related factors. Socioeconomic factors included age (30s, 40s, 50s, 60s and older), sex (male or female), residential region (metropolitan or rural), income level (quintiles), and health insurance type (Medical Aid, NHI self-employed, or NHI employee). Health-related factors included disability status (disabled or nondisabled) and the Charlson Comorbidity Index score. The Charlson Comorbidity Index score was calculated using the International Classification of Diseases, 10th revision, coding algorithm developed by Quan et al. [15]. Cancer-related factors included Surveillance, Epidemiology, and End Results (SEER) summary stage (localized, regional, distant, or unknown), treating hospital location (Seoul, capital region, metropolitan city, or rural area), treating hospital type (tertiary hospital, general hospital, or other hospital), cancer treatment (surgery, surgery with chemotherapy or radiotherapy), types of cancer (gastric, colorectal, liver, lung, breast, or cervical cancer), and year of the cancer diagnosis.

### 2.3. Statistical Analysis

We examined associations between patterns of cancer care utilization and healthcare utilization, medical expenditures, and clinical outcomes. First, we analyzed the distribution of within-region and out-of-region cancer care according to patient characteristics and compared differences using the chi-square test. Next, we compared study outcomes according to cancer care patterns. For continuous variables, including medical expenditures, means and standard deviations were calculated and differences were compared using the *t* test. For categorical outcomes, including delayed cancer treatment, emergency room visits, and mortality, the chi-square test was performed.

Then, multivariable regression analyses were conducted to estimate associations between cancer care utilization patterns and each study outcome. For total medical expenditures, generalized linear models with a log link and gamma distribution were used to estimate relative ratios (RRs) and 95% confidence intervals (CIs). For delayed cancer treatment, logistic regression models were used to estimate odds ratios (ORs) and 95% CIs. For emergency room visits and all-cause mortality, Cox proportional hazards models were used to estimate hazard ratios (HRs) and 95% CIs. The proportional hazards assumption was assessed using Kaplan–Meier survival curves, and differences between groups were evaluated using the log-rank test. All regression models were adjusted for the covariates included in the analysis. All analyses were two-sided, and *p* < 0.05 was considered statistically significant. Models accounted for regional clustering using generalized estimating equations or robust standard errors, as appropriate.

Additionally, we performed sensitivity analyses using a four-category exposure variable combining treatment location and pretreatment care fragmentation. Subgroup analyses were conducted according to cancer stage to assess whether associations between out-of-region cancer care and study outcomes differed by disease stage.

We also conducted additional analyses to assess the robustness and heterogeneity of the main findings. Cancer-specific subgroup analyses were performed separately for each of the six cancer types. To evaluate the potential influence of the 30-day landmark restriction, we repeated the analyses without excluding patients who died or visited the emergency room within 30 days after initial cancer treatment. In addition, propensity score matching (PSM) was performed to reduce baseline differences between patients receiving within-region and out-of-region cancer care. Propensity scores were estimated using age, sex, residential region, income, health insurance type, disability status, CCI score, SEER stage, cancer treatment, types of cancer, and year of cancer diagnosis. Patients were matched using 1:1 greedy nearest-neighbor matching with a caliper of 0.1. Covariate balance after matching was assessed using standardized mean differences, with an absolute standardized mean difference of <0.1 considered indicative of adequate balance. All statistical analyses were performed using SAS version 9.4 (SAS Institute Inc., Cary, NC, USA) and R software (version 4.1.0; R Foundation for Statistical Computing, Vienna, Austria).

## 3. Results

### 3.1. Descriptive Analysis

A total of 303,443 patients were included in the analysis. Of these, 173,451 (57.2%) received their initial cancer treatment outside their residential region and 129,992 (42.8%) received treatment within their residential region (Table 1).

**Figure 2:**
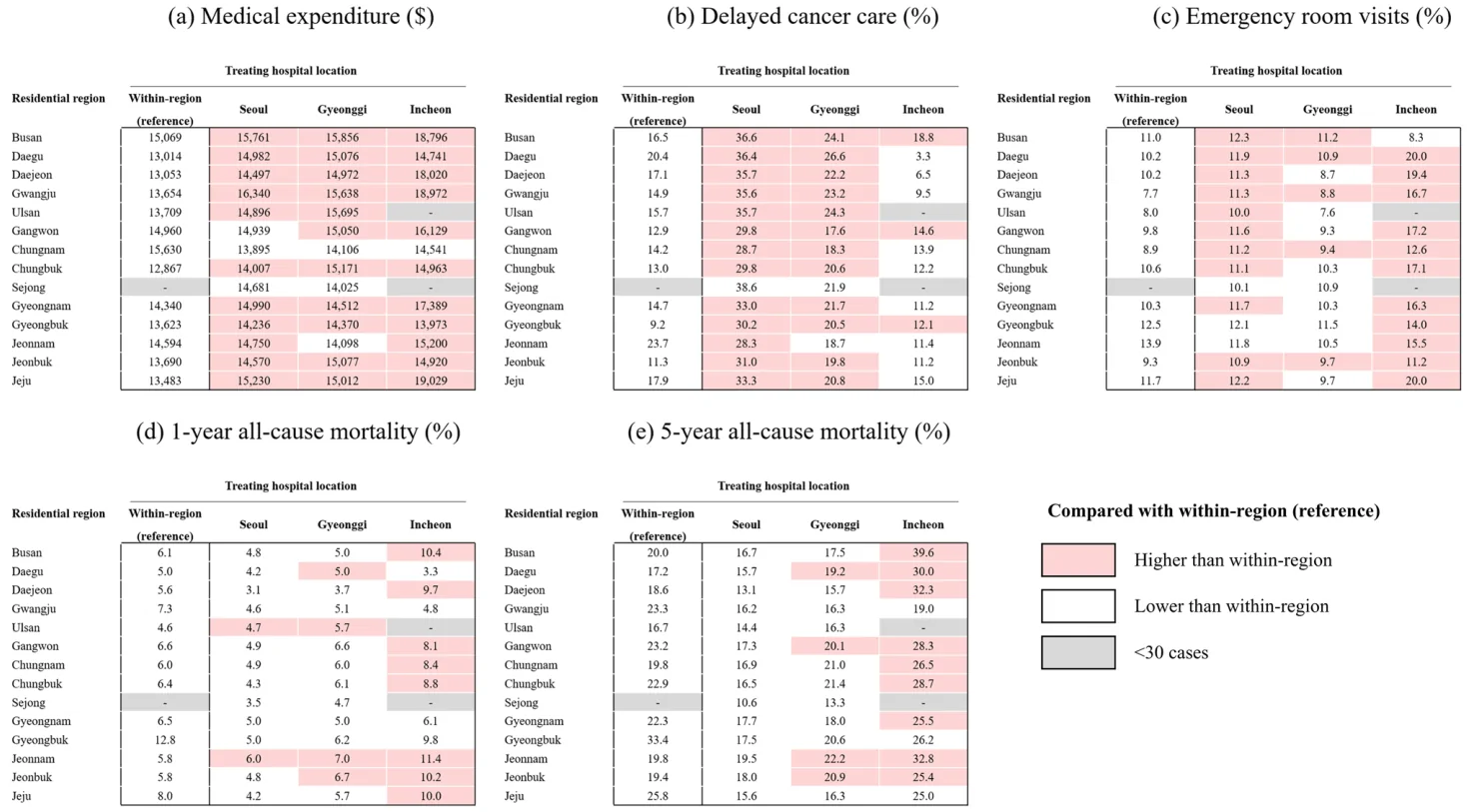
Healthcare utilization and clinical outcomes according to residential and treatment regions. Values represent crude estimates for each residential and treatment region. The within-region column represents patients who received initial cancer treatment within their residential region and serves as the reference. Cells shaded in pink indicate values higher than the corresponding within-region value for each residential region. Gray cells indicate estimates based on fewer than 30 cases. (**a**) Medical expenditure. (**b**) Delayed cancer care. (**c**) Emergency room visits. (**d**) One-year all-cause mortality. (**e**) Five-year all-cause mortality.

Healthcare utilization and clinical outcomes among patients treated within their residential region and those treated in the capital region (Seoul, Gyeonggi, or Incheon) were compared (Figure 2). Overall, patients who received out-of-region treatment in the capital region generally had higher medical expenditures, a greater proportion of delayed cancer treatment, and more frequent emergency room visits compared with those of patients who were treated within their residential region. By contrast, 1-year and 5-year all-cause mortality tended to be lower among patients treated in Seoul or Gyeonggi; however, the patterns were more heterogeneous among those treated in Incheon. Complete comparisons across all residential and treatment regions are presented in Table A1, Table A2, Table A3, Table A4 and Table A5.

Table 2 presents the results of a comparison of healthcare utilization and clinical outcomes according to cancer care utilization patterns. Compared with patients who received within-region cancer care, those who received out-of-region cancer care had significantly higher mean medical expenditures ($14,516 vs. $14,141; *p* < 0.001), a higher proportion of delayed treatment beyond 30 days (27.1% vs. 16.5%; *p* < 0.001), and a higher incidence of emergency room visits after the initial cancer treatment (11.2% vs. 10.4%; *p* < 0.001). By contrast, patients who received out-of-region care experienced lower all-cause mortality than that of patients who received within-region care within 1 year (5.2% vs. 6.0%; *p* < 0.001) and 5 years (18.2% vs. 20.1%; *p* < 0.001) after the initial cancer treatment.

### 3.2. Association of Cancer Care Utilization Patterns with Healthcare Utilization and Clinical Outcomes

Table 3 presents the results of multivariable regression analyses of associations between outcome measures and cancer care utilization patterns. Compared with within-region care, out-of-region care was associated with higher medical expenditures (RR, 1.02; 95% CI, 1.02–1.03), increased odds of delayed treatment (OR, 1.19; 95% CI, 1.15–1.22), and a higher risk of emergency room visits (HR, 1.03; 95% CI, 1.01–1.07). Conversely, out-of-region cancer care was associated with lower 1-year (HR, 0.88; 95% CI, 0.84–0.92) and 5-year (HR 0.93, 95% CI 0.91–0.96) all-cause mortality.

**Figure 3:**
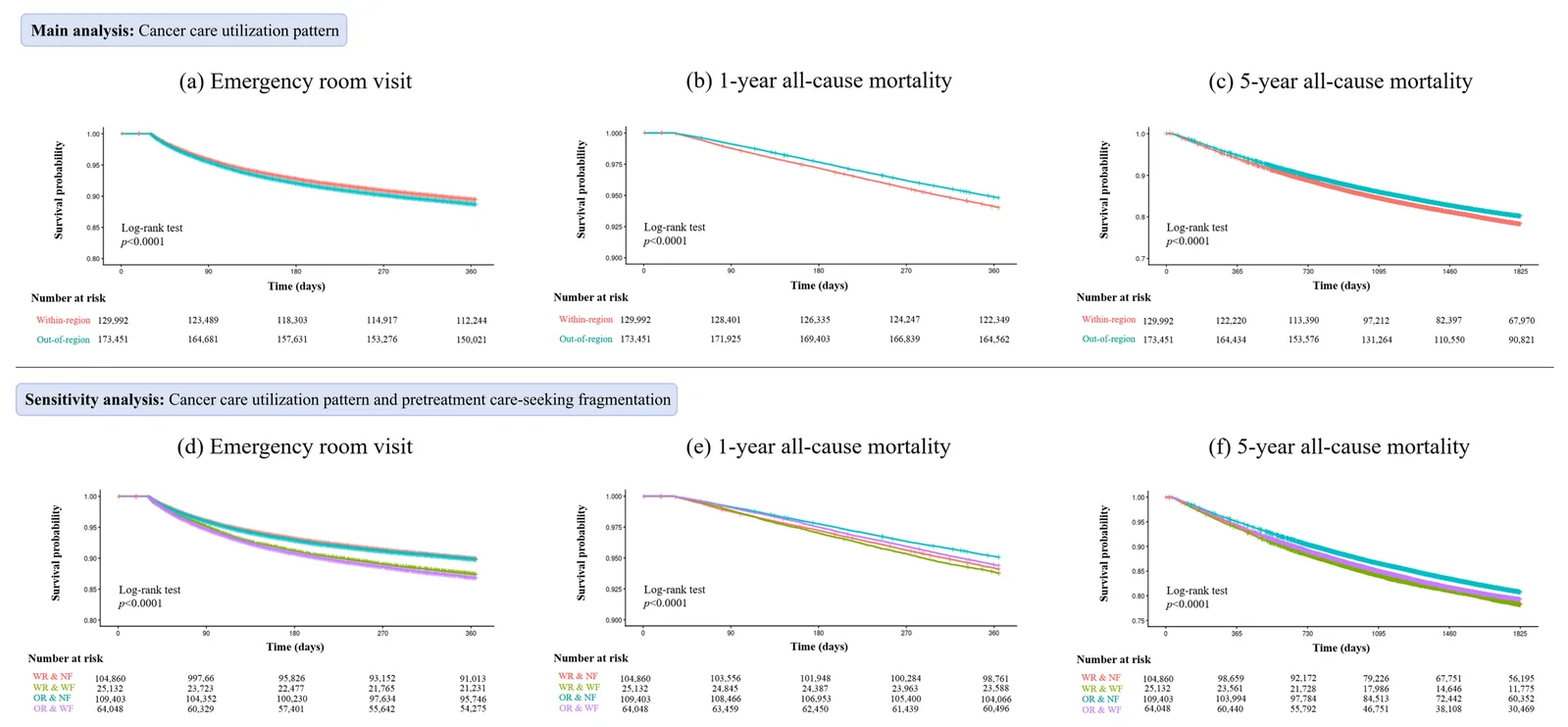
Kaplan–Meier survival curves according to cancer care utilization patterns and pretreatment care-seeking fragmentation. Panels (**a**–**c**) present the main analysis comparing within-region and out-of-region cancer care for emergency room visits, 1-year all-cause mortality, and 5-year all-cause mortality, respectively. Panels (**d**–**f**) present the sensitivity analysis according to cancer care utilization patterns and pretreatment care-seeking fragmentation.

Kaplan–Meier curves were created for emergency room visits and 1-year and 5-year all-cause mortality according to cancer care utilization patterns (Figure 3a–c). The proportional hazards assumption was assessed using Kaplan–Meier survival curves. Additionally, log-rank tests showed significant differences between the within-region and out-of-region groups for emergency room visits and 1-year and 5-year all-cause mortality (all *p* < 0.001). Kaplan–Meier curves were created for the four groups defined by cancer care utilization patterns and pretreatment care-seeking fragmentation (Figure 3d–f). The proportional hazards assumption was assessed using Kaplan–Meier survival curves. Additionally, log-rank tests revealed significant differences among the four groups regarding emergency room visits and 1-year and 5-year all-cause mortality (all *p* < 0.001).

### 3.3. Sensitivity and Subgroup Analysis

Table 4 presents the results of the sensitivity and subgroup analyses. During the sensitivity analysis, multivariable regression analyses were repeated using the four-category exposure variable with the WR/NF group as the reference. Compared with WR/NF, OR/NF (RR, 1.03; 95% CI, 1.02–1.03) and WR/WF (RR, 1.08; 95% CI, 1.07–1.08) were associated with higher medical expenditures, and OR/WF (RR, 1.09; 95% CI, 1.08–1.10) was associated with the highest medical expenditures. Compared with WR/NF, OR/NF (OR, 1.13; 95% CI, 1.10–1.17) and WR/WF (OR, 3.34; 95% CI, 3.23–3.45) were associated with higher odds of delayed treatment, and OR/WF (OR, 4.28; 95% CI, 4.14–4.42) was associated with the highest odds of delayed treatment. Compared with WR/NF, OR/NF (HR 1.04, 95% CI 1.01–1.08) and WR/WF (HR 1.17, 95% CI 1.13–1.22) were associated with a higher risk of emergency room visits, and OR/WF (HR, 1.18; 95% CI, 1.13–1.22) was associated with the highest risk of emergency room visits. Compared with WR/NF, OR/NF (HR, 0.89; 95% CI, 0.84–0.93) was associated with a lower risk of mortality and WR/WF (HR, 1.14; 95% CI, 1.07–1.20) was associated with a higher risk of mortality. OR/WF was not significantly associated with 1-year all-cause mortality. Compared with WR/NF, OR/NF (HR, 0.93; 95% CI, 0.91–0.96) was associated with a lower risk of 5-year all-cause mortality and WR/WF (HR, 1.05; 95% CI, 1.02–1.09) was associated with a higher risk of 5-year all-cause mortality. OR/WF was not significantly associated with 5-year all-cause mortality.

The results of subgroup analyses according to the Surveillance, Epidemiology, and End Results stage are presented in Table 4. Among patients with localized cancer, OR/NF, WR/WF, and OR/WF were associated with increased medical expenditures and delayed cancer treatment compared with those associated with WR/NF. Additionally, WR/WF and OR/WF were associated with an increased risk of emergency room visits. WR/WF was also associated with higher 1-year and 5-year all-cause mortality, whereas OR/NF was associated with lower 1-year and 5-year all-cause mortality. Among patients with regional cancer, WR/WF and OR/WF were associated with increased medical expenditures compared with those associated with WR/NF. OR/NF, WR/WF, and OR/WF were associated with increased odds of delayed cancer treatment. Additionally, WR/WF and OR/WF were associated with an increased risk of emergency room visits. OR/NF was associated with lower 1-year and 5-year all-cause mortality, whereas WR/WF was associated with higher 5-year all-cause mortality. Among patients with distant cancer, no significant associations with medical expenditures were observed. WR/WF and OR/WF were associated with increased odds of delayed cancer treatment. Additionally, OR/NF, WR/WF, and OR/WF were associated with an increased risk of emergency room visits. WR/WF was associated with higher 1-year all-cause mortality, whereas OR/WF was associated with lower 5-year all-cause mortality. Among patients with an unknown cancer stage, WR/WF and OR/WF were associated with increased medical expenditures and delayed cancer treatment. No significant associations with emergency room visits were observed. OR/WF was associated with higher 1-year and 5-year all-cause mortality.

Additional analyses were conducted to assess the robustness and heterogeneity of the main findings (Table A6). When patients who died or visited the emergency room within 30 days after initial cancer treatment were included, the associations with higher medical expenditures, delayed treatment, and lower mortality remained generally consistent, although the association between out-of-region care and emergency room visits was attenuated. Propensity score matching achieved adequate covariate balance between the within-region and out-of-region groups, with absolute standardized mean differences below 0.1 for all included covariates (Table A7). Propensity score-matched analyses similarly showed consistent associations with medical expenditures, delayed treatment, and mortality, whereas the association between out-of-region care and emergency room visits was not statistically significant. In the corresponding four-category analyses, pretreatment care-seeking fragmentation remained strongly associated with delayed treatment and was also associated with higher medical expenditures and emergency room visits in several comparisons. Cancer-specific analyses showed some heterogeneity across cancer types, but the overall patterns for medical expenditures, delayed treatment, and mortality were generally consistent with the main findings.

## 4. Discussion

In this study, out-of-region cancer care was associated with higher medical expenditures and delayed cancer treatment, while it was associated with lower 1-year and 5-year all-cause mortality. Out-of-region care was also associated with increased risk of emergency room visits in the main analysis. In the combined analysis of treatment location and pretreatment care-seeking fragmentation, adverse healthcare utilization outcomes were generally more pronounced among patients with fragmented care-seeking, particularly those receiving out-of-region care. These patterns were also more pronounced among patients with localized or regional cancer.

The future role of regional cancer care systems in South Korea has been considerably debated [5]. From one perspective, patients with severe diseases such as cancer may benefit from directly seeking care at high-volume tertiary hospitals in the capital region and subsequently transitioning to local hospitals after acute treatment if necessary. From another perspective, the importance of regional cancer care systems in providing a timely diagnosis and initial treatment has been emphasized, and referral to higher-level hospitals has been reserved for patients who require more specialized care beyond that provided locally. These competing perspectives reflect an ongoing policy dilemma regarding whether cancer care should be further centralized or delivered through a regionalized healthcare delivery system [16].

Irrespective of whether cancer care is centralized or regionalized, cancer care remains an essential healthcare service that requires a minimum level of regional self-sufficiency. Regional health systems should be able to provide a timely diagnosis, initiate standard treatment when appropriate, manage treatment-related complications, and coordinate follow-up care [10,12]. Therefore, strengthening the regional cancer care capacity is a prerequisite for ensuring continuity, equity, and resilience in the national cancer care system.

Our findings suggest that out-of-region cancer care involves a complex trade-off. Specifically, it may provide access to high-volume hospitals and specialized cancer services potentially associated with better survival; however, it may also impose additional socioeconomic and logistical burdens on patients and the healthcare delivery system.

Lower mortality associated with out-of-region care may reflect a structural paradox in the current healthcare delivery system. This finding is consistent with previous studies reporting an association between higher hospital or provider volume and better cancer outcomes, including lower mortality [17,18]. Although high-volume tertiary hospitals in the capital region may offer advantages such as more specialized cancer care, this pattern also indicates that regional cancer care systems may require further support to achieve comparable clinical capacity [17,18]. Thus, policies aimed at reducing the patient concentration should not restrict patients’ choices. Rather, they should strengthen regional competitiveness through sustained investment in infrastructure, specialized workforce, multidisciplinary care, and referral coordination. Therefore, our findings should not be interpreted as supporting or opposing the patient concentration in the capital region. Rather, they suggest that the consequences of out-of-region cancer care may depend on how patients access initial treatment. Patients who receive care outside their residential region may visit multiple institutions before deciding where to receive active treatment, resulting in fragmented care-seeking before treatment initiation. This pretreatment care fragmentation may contribute to treatment delays, higher medical expenditures, and increased emergency room visits because of reduced continuity of care [19,20,21]. Lower mortality observed among patients who received out-of-region care may reflect the potential benefits of high-volume specialized cancer care, whereas the higher expenditures, treatment delays, and emergency room visits may reflect the costs of fragmented and geographically distant care.

The results of the sensitivity analysis comprising a four-category exposure variable combining treatment location and pretreatment care fragmentation further support this interpretation. Adverse associations of out-of-region care were more pronounced among patients who experienced fragmented care-seeking before initial treatment, suggesting that the burden of out-of-region cancer care may be amplified when geographic mobility is accompanied by fragmented decision-making during the treatment-seeking process. These findings are consistent with previous studies showing that fragmented or multi-institution cancer care may be associated with higher medical expenditures, differences in survival outcomes, and delays in treatment initiation [19,20,21]. Our findings extend this evidence by suggesting that these adverse healthcare utilization patterns may be more pronounced when fragmented care-seeking is accompanied by out-of-region cancer care. However, pretreatment care-seeking fragmentation in this study was measured using multi-institution hospital visits as a proxy; therefore, it may also capture appropriate referral or second-opinion seeking rather than fragmented care in all cases.

When we repeated analyses according to the cancer stage, associations of out-of-region care and pretreatment care fragmentation with healthcare utilization were more pronounced among patients with localized or regional cancer. Among these patients with earlier-stage disease, fragmented care-seeking before treatment may have contributed to higher medical expenditures, delayed treatment initiation, and increased emergency room visits even though their clinical need for highly specialized or centralized treatment may have been relatively limited. By contrast, among patients with advanced cancer, these associations were less consistent and generally attenuated. This may have been attributable to clinical severity, disease progression, and the need for complex multimodal treatment, which play larger roles in determining healthcare utilization for and outcomes of advanced cancer. Among such patients, the influence of care-seeking patterns or treatment location may be relatively lower than the effect of disease complexity itself.

These stage-specific findings have important implications for cancer care quality and delivery. For patients with earlier-stage cancer, an efficient regional cancer care pathway may reduce unnecessary hospital shopping, treatment delays, and unplanned healthcare utilization. Increased emergency room visits of patients with out-of-region care and pretreatment care fragmentation may indicate disrupted continuity of care. Because cancer treatment is generally delivered through planned treatment pathways, unexpected emergency room visits after treatment initiation may reflect insufficient coordination, delayed management of complications, or gaps in information sharing across institutions [20]. For patients with advanced or highly complex cancer, selective referral to high-volume tertiary hospitals may be necessary; however, such referral should be supported by coordinated information-sharing and referral systems to avoid fragmented care and maintain continuity across institutions.

These findings may also be interpreted in the context of medical arms race-like competition among tertiary hospitals, in which the expansion of high-cost specialized cancer services may further reinforce patient concentration in high-volume hospitals [6,7]. Although such concentrations may be associated with better survival outcomes for some patients, they may also exacerbate regional disparities by increasing the burden on patients who must travel outside their residential regions and weakening the role of regional cancer care systems. Overall, out-of-region cancer care may function as a double-edged phenomenon. Although selective referral to large tertiary hospitals may improve outcomes for patients who require highly specialized treatment, excessive or uncoordinated patient movement may increase inefficiencies and fragmentation in cancer care delivery. Therefore, future cancer care policies should focus on developing patient-centered referral systems that balance specialization, continuity, and efficiency of care.

This study had several limitations related to the nature of the data. First, detailed clinical information and patient conditions could not be fully captured because the CPLD is based on secondary administrative and registry data. Nevertheless, these nationwide population-based data provided strong external validity and representativeness. Therefore, these findings may provide useful evidence for improving cancer care policies and healthcare delivery systems. Second, although we adjusted for disease severity using the cancer stage, treatment type, and hospital characteristics, staging information available in the database was less detailed than that in the conventional TNM staging system. Nevertheless, the nationwide cancer registry provides substantial representativeness by covering the majority of patients with cancer in South Korea. Third, although this study assessed 1-year healthcare utilization outcomes and 1-year and 5-year all-cause mortality, longer-term outcomes may be influenced by additional exogenous factors, including disease progression, comorbidities, subsequent treatment decisions, and changes in treatment patterns over time. Therefore, the findings, particularly those related to long-term mortality, should be interpreted with caution. Fourth, the study population was restricted to non-capital-region residents who underwent surgical treatment within one year after cancer diagnosis. The restriction to surgical patients was applied to reduce heterogeneity in treatment pathways, while the restriction to non-capital-region residents was intended to improve the interpretability of out-of-region cancer care in the context of the geographic concentration of cancer care in the capital region. Therefore, our findings may not be generalizable to capital-region residents or to patients with advanced or unresectable cancers who receive exclusively nonsurgical treatment. Future studies should examine out-of-region cancer care in broader patient populations while considering geographic context and cancer-specific treatment sequences, including nonsurgical treatment pathways. Fifth, although the association with lower mortality remained consistent after PSM, residual confounding due to unmeasured patient characteristics and referral-selection effects cannot be excluded. Therefore, the lower mortality observed among patients receiving out-of-region care should be interpreted cautiously and should not be considered evidence of a causal survival benefit. Sixth, in the Cox proportional hazards analysis of emergency room visits, patients who died before experiencing an emergency room visit were censored. Because death may preclude the occurrence of a subsequent emergency room visit, future studies using competing-risk approaches may provide complementary information on the cumulative incidence of emergency room visits.

## 5. Conclusions

Out-of-region cancer care was associated with higher medical expenditures, delayed cancer treatment, and lower all-cause mortality, while the association with emergency department visits was less consistent across sensitivity analyses. These associations with healthcare utilization were more pronounced when out-of-region care was accompanied by pretreatment care-seeking fragmentation. Patient movement should not be restricted. Cancer care policies should strengthen the regional cancer care capacity and establish coordinated referral, patient navigation, and information-sharing systems that balance access to specialized treatment with continuity, efficiency, and regional equity.

## Figures and Tables

**Table 1:** Characteristics of the study population.

Variable	Cancer Care Utilization Pattern				
	Within-Region		Out-of-Region		*p*-Value
	N	%	N	%	
**Total**	129,992	42.8	173,451	57.2	
**Pretreatment care-seeking fragmentation**					
Without	104,860	48.9	109,403	51.1	<0.0001
With	25,132	28.2	64,048	71.8	
**Age**					
30s	4357	34.8	8159	65.2	<0.0001
40s	19,058	40.7	27,749	59.3	
50s	32,758	41.6	45,977	58.4	
60s and older	73,819	44.6	91,566	55.4	
**Sex**					
Male	64,586	43.3	84,684	56.7	<0.0001
Female	65,406	42.4	88,767	57.6	
**Residential** **region**					
Metropolitan	71,188	62.2	43,239	37.8	<0.0001
Rural	58,804	31.1	130,212	68.9	
**Income**					
Q1 (lowest)	33,096	48.8	34,748	51.2	<0.0001
Q2	18,097	46.3	21,013	53.7	
Q3	21,466	44.4	26,882	55.6	
Q4	26,220	41.5	36,944	58.5	
Q5 (highest)	31,113	36.6	53,864	63.4	
**Health insurance type**					
Medical Aid	8665	61.9	5330	38.1	<0.0001
NHI Self-employed	39,780	43.0	52,682	57.0	
NHI Employee	81,547	41.4	115,439	58.6	
**Disability**					
Non-disabled	114,666	42.3	156,469	57.7	<0.0001
Disabled	15,326	47.4	16,982	52.6	
**CCI**					
0	94,341	42.6	126,917	57.4	<0.0001
1	14,815	44.0	18,871	56.0	
≥2	20,836	43.0	27,663	57.0	
**SEER stage**					
Localized	72,755	43.6	94,112	56.4	<0.0001
Regional	46,291	41.9	64,172	58.1	
Distant	7015	38.2	11,359	61.8	
Unknown	3931	50.8	3808	49.2	
**Treating hospital location**					
Seoul			101,251	100.0	<0.0001
Capital			17,548	100.0	
Metropolitan	71,188	64.8	38,647	35.2	
Rural	58,804	78.6	16,005	21.4	
**Treating hospital type**					
Tertiary	81,908	36.5	142,639	63.5	<0.0001
General	44,492	60.6	28,949	39.4	
Other	3592	65.9	1863	34.2	
**Cancer treatment**					
Surgery	75,288	44.2	94,908	55.8	<0.0001
Surgery with chemo or radiotherapy	54,704	41.1	78,543	59.0	
**Types of cancer**					
Gastric	41,242	44.4	51,689	55.6	<0.0001
Colorectal	32,239	46.1	37,686	53.9	
Liver	11,868	36.4	20,725	63.6	
Lung	10,210	34.1	19,720	65.9	
Breast	31,103	43.7	40,074	56.3	
Cervical	3330	48.4	3557	51.7	
**1-year all cause death**					
Survivor	122,223	42.6	164,440	57.4	<0.0001
Died	7769	46.3	9011	53.7	
**Year of cancer diagnosis**					
2012	14,143	43.2	18,570	56.8	<0.0001
2013	14,779	43.5	19,232	56.6	
2014	14,588	43.1	19,275	56.9	
2015	14,538	43.3	19,057	56.7	
2016	15,341	43.1	20,280	56.9	
2017	14,999	42.8	20,070	57.2	
2018	14,553	42.0	20,141	58.1	
2019	13,627	41.8	18,963	58.2	
2020	13,424	42.9	17,863	57.1	

NHI, National Health Insurance; SEER, Surveillance, Epidemiology, and End Results; CCI, Charlson Comorbidity Index. The shaded cells indicate categories not applicable to within-region care because the study population included only non-capital-region residents.

**Table 2:** Characteristics of the study participants according to outcome measures.

Variables	Medical Expenditure ($)			Delayed Cancer Treatment			Emergency Room Visit			1-Year All-Cause Mortality			5-Year All-Cause Mortality		
	Mean	±SD	*p*-Value	N	%	*p*-Value	N	%	*p*-Value	N	%	*p*-Value	N	%	*p*-Value
**Total**	14,355	12,212		68,506	22.6		33,005	10.9		16,780	5.5		57,744	19.0	
**Cancer care utilization pattern**															
Within-region	14,141	11,639	<0.0001	21,442	16.5	<0.0001	13,556	10.4	<0.0001	7769	6.0	<0.0001	26,111	20.1	<0.0001
Out-of-region	14,516	12,623		47,064	27.1		19,449	11.2		9011	5.2		31,633	18.2	
**Pretreatment care-seeking fragmentation**															
Without	13,651	11,737	<0.0001	31,444	14.7	<0.0001	21,497	10.0	<0.0001	11,605	5.4	<0.0001	40,640	19.0	0.18
With	16,048	13,131		37,062	41.6		11,508	12.9		5175	5.8		17,104	19.2	
**Age**															
30s	16,478	11,870	<0.0001	3347	26.7	<0.0001	1712	13.7	<0.0001	364	2.9	<0.0001	1382	11.0	<0.0001
40s	15,930	12,192		11,348	24.2		4741	10.1		1255	2.7		4636	9.9	
50s	15,372	13,175		17,860	22.7		8654	11.0		3208	4.1		11,273	14.3	
60s and older	13,265	11,643		35,951	21.7		17,898	10.8		11,953	7.2		40,453	24.5	
**Sex**															
Male	13,662	13,249	<0.0001	33,620	22.5	<0.0001	18,048	12.1	<0.0001	11,949	8.0	<0.0001	38,655	25.9	<0.0001
emale	15,027	11,076		34,886	22.6		14,957	9.7		4831	3.1		19,089	12.4	
**Reside** **ntial** **region**															
Metropolitan	14,526	12,351		25,973	22.7		12,430	10.9		5904	5.2		20,403	17.8	
Rural	14,252	12,126		42,533	22.5		20,575	10.9		10,876	5.8		37,341	19.8	
**Income**			<0.0001			0.4897			<0.0001			<0.0001			<0.0001
Q1 (lowest)	14,843	12,342	<0.0001	15,150	22.3	0.2107	7846	11.6	0.8470	4328	6.4	<0.0001	14,295	21.1	<0.0001
Q2	15,053	12,294		8895	22.7		4330	11.1		2022	5.2		7061	18.1	
Q3	14,761	12,313		11,034	22.8		5354	11.1		2554	5.3		9016	18.7	
Q4	14,291	12,390		14,353	22.7		6972	11.0		3355	5.3		11,588	18.4	
Q5 (highest)	13,462	11,820		19,074	22.5		8503	10.0		4521	5.3		15,784	18.6	
**Health insurance type**															
Medical Aid	14,099	11,516		2702	19.3		1748	12.5		1396	10.0		4387	31.4	
NHI Self-Employed	14,888	12,464		20,874	22.6		10,629	11.5		5290	5.7		18,003	19.5	
NHI Employee	14,124	12,133		44,930	22.8		20,628	10.5		10,094	5.1		35,354	18.0	
**Disability**															
Non-disabled	14,326	11,860	<0.0001	61,435	22.7	<0.0001	28,993	10.7	<0.0001	13,931	5.1	<0.0001	48,428	17.9	<0.0001
Disabled	14,604	14,843		7071	21.9		4012	12.4		2849	8.8		9316	28.8	
**CCI score**															
0	13,770	11,418	0.0012	48,601	22.0	0.0017	21,430	9.7	<0.0001	10,342	4.7	<0.0001	35,360	16.0	<0.0001
1	15,515	11,652		8400	24.9		4689	13.9		2250	6.7		8172	24.3	
≥2	16,222	15,430		11,505	23.7		6886	14.2		4188	8.6		14,212	29.3	
**SEER Stage**															
Localized	10,680	9758	<0.0001	35,320	21.2	<0.0001	9605	5.8	<0.0001	3446	2.1	<0.0001	16,104	9.7	<0.0001
Regional	17,819	12,154		26,314	23.8		16,494	14.9		7153	6.5		26,474	24.0	
Distant	26,722	16,763		4802	26.1		5939	32.3		5333	29.0		12,747	69.4	
Unknown	14,812	13,891		2070	26.8		967	12.5		848	11.0		2419	31.3	
**Treating hospital location**															
Seoul	14,710	13,066	<0.0001	32,320	31.9	<0.0001	11,686	11.5	<0.0001	4799	4.7	<0.0001	17,093	16.9	<0.0001
Capital	14,806	12,266		3407	19.4		1849	10.5		1103	6.3		3646	20.8	
Metropolitan	13,878	11,735		20,217	18.4		11,185	10.2		6262	5.7		21,258	19.4	
Rural	14,471	11,655		12,562	16.8		8285	11.1		4616	6.2		15,747	21.1	
**Treating hospital type**															
Tertiary	14,305	12,277	<0.0001	56,590	25.2	<0.0001	25,006	11.1	<0.0001	11,452	5.1	<0.0001	40,547	18.1	<0.0001
General	14,437	12,145		11,567	15.8		7846	10.7		5134	7.0		16,486	22.5	
Other	15,331	10,264		349	6.4		153	2.8		194	3.6		711	13.0	
**Cancer treatment**															
Surgery	10,073	9588	<0.0001	35,987	21.1	<0.0001	10,410	6.1	<0.0001	6983	4.1	<0.0001	24,698	14.5	<0.0001
Surgery with chemo or radiotherapy	19,825	12,995		32,519	24.4		22,595	17.0		9797	7.4		33,046	24.8	
**Types of Cancer**															
Gastric	9123	8231	<0.0001	20,200	21.7	<0.0001	7802	8.4	<0.0001	4344	4.7	<0.0001	15,284	16.5	<0.0001
Colorectal	16,227	12,133		13,181	18.9		8024	11.5		3697	5.3		15,004	21.5	
Liver	16,693	18,756		7799	23.9		6174	18.9		5361	16.5		15,004	46.0	
Lung	16,199	12,401		7444	24.9		4136	13.8		2744	9.2		8293	27.7	
Breast	17,650	10,455		18,240	25.6		6257	8.8		501	0.7		3433	4.8	
Cervical	12,829	11,815		1642	23.8		612	8.9		133	1.9		726	10.5	
**1-year all cause death**															
Survivor	13,774	11,435	<0.0001												
Died	24,283	18,939													
**Year of cancer diagnosis**															
2012	11,744	10,004	<0.0001	4855	14.8	<0.0001	3879	11.9	<0.0001	2119	6.5	<0.0001	7311	22.4	<0.0001
2013	11,689	9887		5216	15.3		4000	11.8		2125	6.3		7428	21.8	
2014	12,618	11,094		5548	16.4		3922	11.6		2088	6.2		7261	21.4	
2015	13,495	11,621		6361	18.9		3682	11.0		1865	5.6		6983	20.8	
2016	14,038	12,091		7988	22.4		3746	10.5		1866	5.2		7070	19.9	
2017	14,702	12,673		8733	24.9		3917	11.2		1834	5.2		6922	19.7	
2018	15,777	12,638		10,027	28.9		3827	11.0		1721	5.0		6244	18.0	
2019	17,439	13,651		10,469	32.1		3256	10.0		1664	5.1		4995	15.3	
2020	17,972	13,975		9309	29.8		2776	8.9		1498	4.8		3530	11.3	

NHI, National Health Insurance; SEER, Surveillance, Epidemiology, and End Results; CCI, Charlson Comorbidity Index. The shaded cells indicate variables that were not included as covariates in the corresponding outcome models.

**Table 3:** Results of the multivariable regression analysis.

Variables	Outcomes														
	Medical Expenditure			Delayed Cancer Treatment			Emergency Room Visit			1-Year All-Cause Mortality			5-Year All-Cause Mortality		
	RR	95% CI		OR	95% CI		HR	95% CI		HR	95% CI		HR	95% CI	
		Lower	Upper		Lower	Upper		Lower	Upper		Lower	Upper		Lower	Upper
**Cancer care utilization pattern**															
Within-region	1.00			1.00			1.00			1.00			1.00		
Out-of-region	1.02	1.02	1.03	1.19	1.15	1.22	1.03	1.01	1.07	0.88	0.84	0.92	0.93	0.91	0.96
**Pretreatment care-seeking fragmentation**															
No	1.00			1.00			1.00			1.00			1.00		
Yes	1.07	1.06	1.07	3.63	3.56	3.70	1.14	1.12	1.17	1.11	1.07	1.15	1.05	1.03	1.07
**Age**															
30s	1.00			1.00			1.00			1.00			1.00		
40s	0.99	0.98	1.01	0.95	0.91	1.01	0.75	0.71	0.79	0.85	0.75	0.95	0.82	0.77	0.87
50s	0.99	0.98	0.99	0.95	0.90	0.99	0.75	0.71	0.79	0.88	0.79	0.98	0.87	0.82	0.92
60s and older	0.91	0.90	0.92	1.02	0.97	1.07	0.80	0.76	0.84	1.47	1.33	1.64	1.50	1.42	1.59
**Sex**															
Male	1.00			1.00			1.00			1.00			1.00		
Female	0.98	0.97	0.98	0.85	0.83	0.87	0.99	0.96	1.02	0.79	0.77	0.82	0.82	0.81	0.84
**Residen** **tial** **region**															
Metropolitan	1.00			1.00			1.00			1.00			1.00		
Rural	0.97	0.97	0.98	0.90	0.88	0.92	0.97	0.95	0.99	1.17	1.13	1.22	1.14	1.12	1.16
**Income**															
Q1 (lowest)	1.00			1.00			1.00			1.00			1.00		
Q2	1.00	0.99	1.01	0.99	0.96	1.03	0.96	0.92	0.99	0.95	0.90	1.01	0.98	0.95	1.01
Q3	0.99	0.99	0.99	0.98	0.95	1.01	0.96	0.92	0.99	0.93	0.88	0.98	0.97	0.95	1.01
Q4	0.98	0.98	0.99	0.95	0.92	0.98	0.97	0.93	1.01	0.91	0.87	0.96	0.93	0.90	0.95
Q5 (highest)	0.95	0.94	0.96	0.89	0.87	0.91	0.93	0.90	0.96	0.99	0.94	1.03	1.01	0.98	1.04
**Health insurance type**															
Medical Aid	1.00			1.00			1.00			1.00			1.00		
NHI Self-Employed	1.07	1.06	1.08	0.98	0.93	1.03	0.99	0.94	1.05	0.79	0.74	0.85	0.76	0.73	0.79
NHI Employee	1.04	1.03	1.05	0.96	0.91	1.01	0.94	0.89	0.99	0.73	0.68	0.78	0.71	0.68	0.73
**Disability**															
Non-disabled	1.00			1.00			1.00			1.00			1.00		
Disabled	1.09	1.08	1.10	1.08	1.05	1.11	1.15	1.11	1.19	1.28	1.22	1.33	1.28	1.25	1.31
**CCI score**	1.05	1.05	1.05	1.10	1.09	1.11	1.21	1.20	1.23	1.13	1.12	1.15	1.12	1.11	1.13
**SEER Stage**															
Localized	1.00			1.00			1.00			1.00			1.00		
Regional	1.41	1.40	1.41	1.09	1.06	1.11	2.26	2.19	2.32	3.77	3.61	3.93	3.00	2.94	3.06
Distant	1.92	1.90	1.93	1.20	1.15	1.25	4.85	4.68	5.03	16.90	16.12	17.71	12.40	12.08	12.73
Unknown	1.14	1.12	1.15	1.59	1.50	1.68	1.55	1.45	1.66	3.18	2.94	3.43	2.25	2.15	2.35
**Treating hospital location**															
Seoul	1.00			1.00			1.00			1.00			1.00		
Capital	1.06	1.05	1.07	0.59	0.57	0.62	0.99	0.94	1.04	1.27	1.18	1.36	1.21	1.17	1.26
Metropolitan	1.00	0.99	1.01	0.70	0.68	0.72	1.08	1.04	1.11	1.43	1.36	1.50	1.38	1.34	1.41
Rural	1.09	1.08	1.10	0.64	0.62	0.66	1.17	1.12	1.21	1.30	1.22	1.38	1.31	1.27	1.35
**Treating hospital type**															
Tertiary	1.00			1.00			1.00			1.00			1.00		
General	1.04	1.03	1.04	0.81	0.79	0.83	0.94	0.92	0.97	1.22	1.17	1.26	1.12	1.09	1.14
Other	0.97	0.95	0.98	0.19	0.17	0.21	0.26	0.22	0.30	1.54	1.34	1.78	1.22	1.14	1.32
**Cancer treatment**															
Surgery	1.00			1.00			1.00			1.00			1.00		
Surgery with chemo or radiotherapy	1.54	1.53	1.55	0.98	0.95	0.99	2.30	2.24	2.37	1.17	1.13	1.21	1.52	1.49	1.55
**Types of Cancer**															
Gastric	1.00			1.00			1.00			1.00			1.00		
Colorectal	1.31	1.30	1.32	0.77	0.75	0.80	0.71	0.69	0.74	0.54	0.52	0.57	0.62	0.60	0.63
Liver	1.32	1.31	1.33	0.83	0.80	0.86	1.06	1.01	1.10	2.62	2.50	2.76	2.38	2.31	2.45
Lung	1.44	1.43	1.45	1.09	1.05	1.13	1.06	1.02	1.10	1.22	1.16	1.28	1.12	1.09	1.15
Breast	1.41	1.40	1.42	1.25	1.21	1.29	0.63	0.60	0.66	0.18	0.17	0.20	0.27	0.26	0.28
Cervical	1.16	1.15	1.18	1.38	1.29	1.47	0.81	0.74	0.88	0.54	0.45	0.64	0.71	0.66	0.76
**1-year all-cause death**															
Survivor	1.00														
Died	1.52	1.51	1.53												
**Year of cancer diagnosis**	1.06	1.06	1.06	1.13	1.13	1.14	0.97	0.97	0.98	0.96	0.96	0.97	0.98	0.97	0.98

RR, relative risk; OR, odds ratio; HR, hazard ratio; CI, confidence interval; NHI, National Health Insurance; SEER, Surveillance, Epidemiology, and End Results; CCI, Charlson Comorbidity Index. The shaded cells indicate variables that were not included as covariates in the corresponding outcome models.

**Table 4:** Results of the sensitivity and subgroup analyses.

Variables	Outcomes														
	Medical Expenditure			Delayed Cancer Treatment			Emergency Room Visit			1-Year All-Cause Mortality			5-Year All-Cause Mortality		
	RR	95% CI		OR	95% CI		HR	95% CI		HR	95% CI		HR	95% CI	
		Lower	Upper		Lower	Upper		Lower	Upper		Lower	Upper		Lower	Upper
**Total participants**															
** Treatment location and fragmented care**															
WR/NF	1.00			1.00			1.00			1.00			1.00		
OR/NF	1.03	1.02	1.03	1.13	1.10	1.17	1.04	1.01	1.08	0.89	0.84	0.93	0.93	0.91	0.96
WR/WF	1.08	1.07	1.08	3.34	3.23	3.45	1.17	1.13	1.22	1.14	1.07	1.20	1.05	1.02	1.09
OR/WF	1.09	1.08	1.10	4.28	4.14	4.42	1.18	1.13	1.22	0.97	0.92	1.02	0.98	0.95	1.01
**Localized stage**															
** Cancer care utilization pattern**															
Within-region	1.00			1.00			1.00			1.00			1.00		
Out-of-region	1.04	1.03	1.05	1.27	1.22	1.32	1.02	0.97	1.08	0.82	0.74	0.90	0.92	0.88	0.96
**Treatment location and fragmented care**															
WR/NF	1.00			1.00			1.00			1.00			1.00		
OR/NF	1.05	1.04	1.05	1.20	1.15	1.25	1.03	0.97	1.09	0.83	0.75	0.93	0.92	0.88	0.97
WR/WF	1.13	1.12	1.14	3.22	3.07	3.37	1.24	1.15	1.34	1.31	1.16	1.48	1.10	1.04	1.18
OR/WF	1.15	1.14	1.16	4.61	4.41	4.82	1.25	1.17	1.34	0.99	0.87	1.13	1.01	0.95	1.07
**Regional stage**															
** Cancer care utilization pattern**															
Within-region	1.00			1.00			1.00			1.00			1.00		
Out-of-region	1.01	1.00	1.01	1.13	1.08	1.18	1.03	0.99	1.08	0.84	0.78	0.90	0.93	0.90	0.96
**Treatment location and fragmented care**															
WR/NF	1.00			1.00			1.00			1.00			1.00		
OR/NF	1.00	0.99	1.01	1.09	1.03	1.15	1.05	1.00	1.10	0.83	0.77	0.90	0.93	0.89	0.97
WR/WF	1.01	1.01	1.02	3.36	3.19	3.54	1.15	1.09	1.22	1.07	0.98	1.16	1.06	1.02	1.11
OR/WF	1.02	1.01	1.03	4.02	3.81	4.24	1.15	1.09	1.21	0.92	0.85	1.00	0.98	0.94	1.03
**Distant stage**															
** Cancer care utilization pattern**															
Within-region	1.00			1.00			1.00			1.00			1.00		
Out-of-region	1.01	0.98	1.03	1.01	0.90	1.13	1.08	1.01	1.18	0.94	0.86	1.02	0.94	0.89	0.99
**Treatment location and fragmented care**															
WR/NF	1.00			1.00			1.00			1.00			1.00		
OR/NF	1.01	0.98	1.03	1.01	0.89	1.16	1.13	1.04	1.24	0.97	0.89	1.06	0.95	0.90	1.01
WR/WF	1.01	0.98	1.04	4.58	4.03	5.20	1.23	1.11	1.35	1.14	1.03	1.26	1.02	0.96	1.09
OR/WF	1.01	0.98	1.04	4.64	4.07	5.28	1.21	1.10	1.32	0.97	0.88	1.07	0.91	0.86	0.97
**Unknown stage**															
** Cancer care utilization pattern**															
Within-region	1.00			1.00			1.00			1.00			1.00		
Out-of-region	0.97	0.92	1.02	1.00	0.82	1.22	0.91	0.72	1.16	1.22	0.98	1.53	1.00	0.87	1.15
**Treatment location and fragmented care**															
WR/NF	1.00			1.00			1.00			1.00			1.00		
OR/NF	0.95	0.90	1.00	0.93	0.76	1.15	0.85	0.67	1.09	1.20	0.94	1.52	0.99	0.85	1.14
WR/WF	1.12	1.06	1.19	3.40	2.81	4.11	0.93	0.72	1.21	1.17	0.90	1.53	1.14	0.97	1.34
OR/WF	1.17	1.10	1.25	4.00	3.20	5.00	1.09	0.83	1.42	1.54	1.18	2.01	1.21	1.02	1.43

RR, relative risk; OR, odds ratio; HR, hazard ratio; CI, confidence interval; WR/NF, within-region care without pretreatment care-seeking fragmentation; OR/NF, out-of-region care without pretreatment care-seeking fragmentation; WR/WF, within-region care with pretreatment care-seeking fragmentation; OR/WF, out-of-region care with pretreatment care-seeking fragmentation.

## Data Availability

The data that support the findings of this study are available from the Cancer Public Library Database (CPLD) of the Korea Clinical Data Utilization Network for Research Excellence (K-CURE). However, these data were used under license for the current study and are not publicly available. The data may be accessed through the K-CURE data application process, subject to approval by the relevant authority. Further information is available at https://k-cure.mohw.go.kr (accessed on 20 July 2026).

## Appendix A

**Table A1:** Medical expenditures according to residential and treatment regions.

(Unit: %)																		
Cluster		Treating Hospital Location																
		Capital Region			Busan	Daegu	Daejeon	Gwangju	Ulsan	Gangwon	Chung nam	Chung buk	Sejong	Gyeong nam	Gyeong buk	Jeonnam	Jeonbuk	Jeju
		Seoul	Gyeonggi	Incheon														
**Residential region**	**Busan**	15,761	15,856	18,796	**15,069**	14,325	-	-	13,771	-	-	-	-	14,360	-	-	-	-
	**Daegu**	14,982	15,076	14,741	15,141	**13,014**	14,777	-	-	-	-	-	-	13,827	-	-	-	-
	**Daejeon**	14,497	14,972	18,020	-	15,099	**13,053**	-	-	-	18,413	-	-	-	-	-	-	-
	**Gwangju**	16,340	15,638	18,972	-	-	-	**13,654**	-	-	-	-	-	-	-	16,003	14,720	-
	**Ulsan**	14,896	15,695	-	17,204	14,938	-	-	**13,709**	-	-	-	-	14,005	-	-	-	-
	**Gangwon**	14,939	15,050	16,129	16,788	12,029	12,961	-	-	**14,960**	15,619	-	-	-	-	-	-	-
	**Chungnam**	13,895	14,106	14,541	19,066	12,601	13,073	-	-	-	**15,630**	11,796	-	-	-	-	12,979	-
	**Chungbuk**	14,007	15,171	14,963	16,931	14,373	12,799	-	-	13,841	17,197	**12,867**	-	-	-	-	-	-
	**Sejong**	14,681	14,025	-	-	-	13,658	-	-	-	14,023	13,338	-	-	-	-	-	-
	**Gyeongnam**	14,990	14,512	17,389	15,434	12,836	13,075	-	14,395	-	14,018	-	-	**14,340**	-	17,093	14,800	-
	**Gyeongbuk**	14,236	14,370	13,973	15,047	12,298	12,992	-	12,851	15,070	16,363	12,650	-	13,740	**13,623**	-	-	-
	**Jeonnam**	14,750	14,098	15,200	14,357	14,187	11,886	12,929	-	-	13,652	-	-	13,460	-	**14,594**	13,533	-
	**Jeonbuk**	14,570	15,077	14,920	14,016	11,904	13,692	13,005	-	-	16,353	-	-	-	-	15,434	**13,690**	-
	**Jeju**	15,230	15,012	19,029	16,426	12,566	-	-	-	-	-	-	-	-	-	17,465	-	**13,483**

Bold values represent the mean health expenditures of patients who received treatment within their residential region (within-region). Cells highlighted in red indicate higher mean health expenditures compared with the corresponding within-region values. Gray cells indicate combinations with fewer than 30 patients (approximately 0.01% of the total study population). Corresponding values are not presented.

**Table A2:** Delayed cancer care according to residential region and treatment region.

(Unit: %)																		
**Cluster**		Treating Hospital Location																
		Capital Region			Busan	Daegu	Daejeon	Gwangju	Ulsan	Gangwon	Chung nam	Chung buk	Sejong	Gyeong nam	Gyeong buk	Jeonnam	Jeonbuk	Jeju
		Seoul	Gyeonggi	Incheon														
**Residential region**	**Busan**	36.6	24.1	18.8	**16.5**	27.3	-	-	16.3	-	-	-	-	26.4	-	-	-	-
	**Daegu**	36.4	26.6	3.3	16.4	**20.4**	20.6	-	-	-	-	-	-	11.8	-	-	-	-
	**Daejeon**	35.7	22.2	6.5	-	18.8	**17.1**	-	-	-	16.2	-	-	-	-	-	-	-
	**Gwangju**	35.6	23.2	9.5	-	-	-	**14.9**	-	-	-	-	-	-	-	23.2	10.5	-
	**Ulsan**	35.7	24.3	-	18.3	21.4	-	-	**15.7**	-	-	-	-	26.6	-	-	-	-
	**Gangwon**	29.8	17.6	14.6	25.9	28.6	18.2	-	-	**12.9**	20.5	-	-	-	-	-	-	-
	**Chungnam**	28.7	18.3	13.9	30.4	26.1	15.3	-	-	-	**14.2**	20.0	-	-	-	-	14.8	-
	**Chungbuk**	29.8	20.6	12.2	22.5	31.0	18.8	-	-	17.7	15.1	**13.0**	-	-	-	-	-	-
	**Sejong**	38.6	21.9	-	-	-	13.1	-	-	-	15.4	14.4	-	-	-	-	-	-
	**Gyeongnam**	33.0	21.7	11.2	15.3	20.0	16.9	-	15.5	-	20.4	-	-	**14.7**	-	24.0	9.4	-
	**Gyeongbuk**	30.2	20.5	12.1	15.0	26.3	20.7	-	16.2	14.4	22.4	9.3	-	26.7	**9.2**	-	-	-
	**Jeonnam**	28.3	18.7	11.4	18.3	21.4	15.6	14.0	-	-	18.0	-	-	23.6	-	**23.7**	11.1	-
	**Jeonbuk**	31.0	19.8	11.2	17.8	22.0	13.6	19.2	-	-	16.1	-	-	-	-	26.2	**11.3**	-
	**Jeju**	33.3	20.8	15.0	20.0	32.4	-	-	-	-	-	-	-	-	-	25.7	-	**17.9**

Bold values represent delayed cancer care among patients who received treatment within their residential regions (within-region). Cells highlighted in red indicate higher proportions of delayed cancer care compared with the corresponding within-region values. Gray cells indicate combinations with fewer than 30 patients (approximately 0.01% of the total study population). Corresponding values are not presented.

**Table A3:** Emergency room visits according to residential region and treatment region.

(Unit: %)																		
Cluster		Treating Hospital Location																
		Capital Region			Busan	Daegu	Daejeon	Gwangju	Ulsan	Gangwon	Chung nam	Chung buk	Sejong	Gyeong nam	Gyeong buk	Jeonnam	Jeonbuk	Jeju
		Seoul	Gyeonggi	Incheon														
**Residential region**	**Busan**	12.3	11.2	8.3	**11.0**	12.1	-	-	7.8	-	-	-	-	12.4	-	-	-	-
	**Daegu**	11.9	10.9	20.0	10.9	**10.2**	8.8	-	-	-	-	-	-	8.8	-	-	-	-
	**Daejeon**	11.3	8.7	19.4	-	18.8	**10.2**	-	-	-	8.1	-	-	-	-	-	-	-
	**Gwangju**	11.3	8.8	16.7	-	-	-	**7.7**	-	-	-	-	-	-	-	14.2	5.3	-
	**Ulsan**	10.0	7.6	-	9.6	8.3	-	-	**8.0**	-	-	-	-	10.5	-	-	-	-
	**Gangwon**	11.6	9.3	17.2	14.8	9.5	25.0	-	-	**9.8**	15.4	-	-	-	-	-	-	-
	**Chungnam**	11.2	9.4	12.6	6.5	10.9	9.9	-	-	-	**8.9**	8.6	-	-	-	-	10.8	-
	**Chungbuk**	11.1	10.3	17.1	15.0	18.3	10.7	-	-	9.4	12.3	**10.6**	-	-	-	-	-	-
	**Sejong**	10.1	10.9	-	-	-	9.3	-	-	-	7.4	8.0	-	-	-	-	-	-
	**Gyeongnam**	11.7	10.3	16.3	9.9	9.0	15.3	-	10.5	-	10.2	-	-	**10.3**	-	10.0	15.6	-
	**Gyeongbuk**	12.1	11.5	14.0	9.1	10.3	14.7	-	7.4	7.6	14.5	5.6	-	10.1	**12.5**	-	-	-
	**Jeonnam**	11.8	10.5	15.5	10.7	11.9	10.4	10.9	-	-	9.8	-	-	10.9	-	**13.9**	7.4	-
	**Jeonbuk**	10.9	9.7	11.2	2.2	7.3	12.1	6.4	-	-	8.9	-	-	-	-	12.6	**9.3**	-
	**Jeju**	12.2	9.7	20.0	8.8	11.8	-	-	-	-	-	-	-	-	-	11.4	-	**11.7**

Bold values represent emergency room visits among patients who received treatment within their residential regions (within-region). The cells highlighted in red indicate higher proportions of emergency room visits compared with the corresponding within-region values. Gray cells indicate combinations with fewer than 30 patients (approximately 0.01% of the total study population). Corresponding values are not presented.

**Table A4:** One-year all-cause mortality according to residential region and treatment region.

(Unit: %)																		
Cluster		Treating Hospital Location																
		Capital Region			Busan	Daegu	Daejeon	Gwangju	Ulsan	Gangwon	Chung nam	Chung buk	Sejong	Gyeong nam	Gyeong buk	Jeonnam	Jeonbuk	Jeju
		Seoul	Gyeonggi	Incheon														
**Residential region**	**Busan**	4.8	5.0	10.4	**6.1**	6.8	-	-	4.7	-	-	-	-	5.9	-	-	-	-
	**Daegu**	4.2	5.0	3.3	7.3	**5.0**	8.8	-	-	-	-	-	-	2.9	-	-	-	-
	**Daejeon**	3.1	3.7	9.7	-	12.5	**5.6**	-	-	-	8.1	-	-	-	-	-	-	-
	**Gwangju**	4.6	5.1	4.8	-	-	-	**7.3**	-	-	-	-	-	-	-	3.7	7.9	-
	**Ulsan**	4.7	5.7	-	4.4	3.6	-	-	**4.6**	-	-	-	-	5.3	-	-	-	-
	**Gangwon**	4.9	6.6	8.1	7.4	4.8	6.8	-	-	**6.6**	12.8	-	-	-	-	-	-	-
	**Chungnam**	4.9	6.0	8.4	4.3	4.3	7.6	-	-	-	**6.0**	10.0	-	-	-	-	8.1	-
	**Chungbuk**	4.3	6.1	8.8	12.5	7.0	6.2	-	-	8.1	6.2	**6.4**	-	-	-	-	-	-
	**Sejong**	3.5	4.7	-	-	-	5.7	-	-	-	10.3	8.6	-	-	-	-	-	-
	**Gyeongnam**	5.0	5.0	6.1	5.3	6.1	11.9	-	7.9	-	4.1	-	-	**6.5**	-	2.0	6.3	-
	**Gyeongbuk**	5.0	6.2	9.8	7.2	5.0	10.0	-	6.9	4.7	11.8	11.1	-	5.7	**12.8**	-	-	-
	**Jeonnam**	6.0	7.0	11.4	8.9	9.5	5.2	8.9	-	-	14.8	-	-	8.2	-	**5.8**	5.9	-
	**Jeonbuk**	4.8	6.7	10.2	6.7	2.4	8.5	8.7	-	-	8.9	-	-	-	-	4.1	**5.8**	-
	**Jeju**	4.2	5.7	10.0	3.8	2.9	-	-	-	-	-	-	-	-	-	2.9	-	**8.0**

Bold values represent 1-year all-cause mortality among patients who received treatment within their residential regions (within-region). Cells highlighted in red indicate higher proportions of 1-year all-cause mortality compared with the corresponding within-region values. Gray cells indicate combinations with fewer than 30 patients (approximately 0.01% of the total study population). Corresponding values are not presented.

**Table A5:** Five-year all-cause mortality according to residential region and treatment region.

(Unit: %)																		
Cluster		Treating Hospital Location																
		Capital Region			Busan	Daegu	Daejeon	Gwangju	Ulsan	Gangwon	Chung nam	Chung buk	Sejong	Gyeong nam	Gyeong buk	Jeonnam	Jeonbuk	Jeju
		Seoul	Gyeonggi	Incheon														
**Residential region**	**Busan**	16.7	17.5	39.6	**20.0**	20.5	-	-	24.0	-	-	-	-	20.7	-	-	-	-
	**Daegu**	15.7	19.2	30.0	17.3	**17.2**	20.6	-	-	-	-	-	-	14.7	-	-	-	-
	**Daejeon**	13.1	15.7	32.3	-	21.9	**18.6**	-	-	-	16.2	-	-	-	-	-	-	-
	**Gwangju**	16.2	16.3	19.0	-	-	-	**23.3**	-	-	-	-	-	-	-	15.5	18.4	-
	**Ulsan**	14.4	16.3	-	16.5	14.3	-	-	**16.7**	-	-	-	-	20.3	-	-	-	-
	**Gangwon**	17.3	20.1	28.3	27.8	14.3	18.2	-	-	**23.2**	35.9	-	-	-	-	-	-	-
	**Chungnam**	16.9	21.0	26.5	26.1	19.6	22.4	-	-	-	**19.8**	25.7	-	-	-	-	23.5	-
	**Chungbuk**	16.5	21.4	28.7	32.5	35.2	22.7	-	-	23.7	16.4	**22.9**	-	-	-	-	-	-
	**Sejong**	10.6	13.3	-	-	-	17.6	-	-	-	19.1	24.7	-	-	-	-	-	-
	**Gyeongnam**	17.7	18.0	25.5	18.6	21.4	32.2	-	28.5	-	26.5	-	-	**22.3**	-	20.0	18.8	-
	**Gyeongbuk**	17.5	20.6	26.2	22.0	19.0	26.7	-	24.3	23.7	27.6	25.9	-	21.5	**33.4**	-	-	-
	**Jeonnam**	19.5	22.2	32.8	21.9	21.4	24.7	28.9	-	-	32.8	-	-	25.5	-	**19.8**	19.3	-
	**Jeonbuk**	18.0	20.9	25.4	24.4	26.8	25.4	31.5	-	-	25.0	-	-	-	-	18.8	**19.4**	-
	**Jeju**	15.6	16.3	25.0	18.8	17.6	-	-	-	-	-	-	-	-	-	20.0	-	**25.8**

Bold values represent 5-year all-cause mortality among patients who received treatment within their residential regions (within-region). Cells highlighted in red indicate higher proportions of 5-year all-cause mortality compared with the corresponding within-region values. Gray cells indicate combinations with fewer than 30 patients (approximately 0.01% of the total study population). Corresponding values are not presented.

**Table A6:** Results of additional sensitivity and subgroup analyses.

Variables	Outcomes														
	Medical Expenditure			Delayed Cancer Treatment			Emergency Room Visit			1-Year All-Cause Mortality			5-Year All-Cause Mortality		
	RR	95% CI		OR	95% CI		HR	95% CI		HR	95% CI		HR	95% CI	
		Lower	Upper		Lower	Upper		Lower	Upper		Lower	Upper		Lower	Upper
**Including early events within 30 days**															
** Cancer care utilization pattern**															
Within-region	1.00			1.00			1.00			1.00			1.00		
Out-of-region	1.02	1.02	1.03	1.18	1.15	1.21	1.00	0.98	1.02	0.88	0.85	0.91	0.93	0.91	0.95
** Treatment location and fragmented care**															
WR/NF	1.00			1.00			1.00			1.00			1.00		
OR/NF	1.02	1.02	1.03	1.15	1.11	1.18	0.98	0.96	1.01	0.89	0.85	0.92	0.93	0.91	0.95
WR/WF	1.06	1.05	1.07	3.60	3.49	3.71	0.96	0.93	0.99	1.14	1.08	1.19	1.04	1.01	1.07
OR/WF	1.08	1.07	1.09	4.44	4.30	4.58	1.02	0.99	1.05	0.97	0.92	1.01	0.97	0.94	0.99
**Propensity score matching**															
** Cancer care utilization pattern**															
Within-region	1.00			1.00			1.00			1.00			1.00		
Out-of-region	1.02	1.01	1.02	1.19	1.15	1.23	1.03	0.99	1.07	0.87	0.82	0.91	0.93	0.90	0.95
** Treatment location and fragmented care**															
WR/NF	1.00			1.00			1.00			1.00			1.00		
OR/NF	1.02	1.01	1.03	1.17	1.13	1.21	1.05	1.01	1.10	0.88	0.83	0.93	0.93	0.91	0.96
WR/WF	1.07	1.06	1.08	3.55	3.42	3.68	1.20	1.14	1.25	1.15	1.08	1.22	1.07	1.03	1.11
OR/WF	1.08	1.07	1.09	4.33	4.17	4.50	1.18	1.13	1.24	0.95	0.89	1.02	0.97	0.93	1.01
**Gastric cancer**															
** Cancer care utilization pattern**															
Within-region	1.00			1.00			1.00			1.00			1.00		
Out-of-region	1.05	1.04	1.07	1.39	1.33	1.47	1.02	0.95	1.08	0.86	0.79	0.94	0.94	0.89	0.98
** Treatment location and fragmented care**															
WR/NF	1.00			1.00			1.00			1.00			1.00		
OR/NF	1.07	1.06	1.08	1.36	1.29	1.44	1.03	0.96	1.11	0.87	0.79	0.96	0.97	0.92	1.02
WR/WF	1.15	1.14	1.17	3.26	3.07	3.47	1.18	1.09	1.27	1.09	0.99	1.21	1.10	1.04	1.17
OR/WF	1.16	1.15	1.18	4.72	4.45	5.01	1.16	1.07	1.25	0.91	0.82	1.02	0.95	0.90	1.01
**Colorectal cancer**															
** Cancer care utilization pattern**															
Within-region	1.00			1.00			1.00			1.00			1.00		
Out-of-region	1.00	0.99	1.01	1.14	1.08	1.21	1.13	1.06	1.20	0.90	0.82	0.99	0.92	0.88	0.97
** Treatment location and fragmented care**															
WR/NF	1.00			1.00			1.00			1.00			1.00		
OR/NF	1.00	0.99	1.01	1.10	1.03	1.18	1.19	1.11	1.28	0.93	0.84	1.04	0.92	0.87	0.97
WR/WF	0.98	0.97	0.99	3.61	3.39	3.84	1.37	1.27	1.48	1.09	0.98	1.21	1.01	0.95	1.06
OR/WF	0.98	0.97	0.99	4.33	4.03	4.61	1.37	1.27	1.48	0.90	0.80	1.01	0.93	0.88	0.99
**Lung cancer**															
** Cancer care utilization pattern**															
Within-region	1.00			1.00			1.00			1.00			1.00		
Out-of-region	0.99	0.98	1.01	0.95	0.86	1.05	1.01	0.92	1.10	0.89	0.80	0.99	0.95	0.90	1.02
** Treatment location and fragmented care**															
WR/NF	1.00			1.00			1.00			1.00			1.00		
OR/NF	0.99	0.97	1.01	0.95	0.85	1.06	0.99	0.90	1.09	0.91	0.81	1.02	0.95	0.89	1.02
WR/WF	1.01	0.98	1.04	5.70	4.92	6.61	1.08	0.93	1.25	1.31	1.12	1.53	1.08	0.97	1.20
OR/WF	1.04	1.02	1.06	5.37	4.77	6.04	1.18	1.05	1.32	1.05	0.91	1.21	1.06	0.98	1.16
**Liver cancer**															
** Cancer care utilization pattern**															
Within-region	1.00			1.00			1.00			1.00			1.00		
Out-of-region	1.02	0.99	1.04	1.08	0.99	1.17	0.94	0.86	1.02	0.89	0.82	0.97	0.93	0.89	0.98
** Treatment location and fragmented care**															
WR/NF	1.00			1.00			1.00			1.00			1.00		
OR/NF	1.01	0.99	1.04	1.14	1.04	1.24	0.94	0.86	1.02	0.91	0.83	0.99	0.94	0.89	0.99
WR/WF	1.08	1.04	1.12	5.12	4.57	5.73	1.15	1.03	1.29	1.2	1.07	1.35	1.13	1.05	1.21
OR/WF	1.14	1.11	1.17	4.66	4.23	5.15	1.07	0.98	1.18	1.02	0.92	1.13	1.01	0.96	1.08
**Breast cancer**															
** Cancer care utilization pattern**															
Within-region	1.00			1.00			1.00			1.00			1.00		
Out-of-region	1.03	1.01	1.04	1.14	1.07	1.21	1.03	0.96	1.11	0.75	0.58	0.98	0.93	0.84	1.02
** Treatment location and fragmented care**															
WR/NF	1.00			1.00			1.00			1.00			1.00		
OR/NF	1.03	1.02	1.04	0.99	0.92	1.07	1.05	0.97	1.14	0.72	0.53	0.96	0.95	0.85	1.06
WR/WF	1.06	1.05	1.08	2.76	2.58	2.96	1.08	0.99	1.18	1.12	0.85	1.46	1.12	1.01	1.25
OR/WF	1.08	1.07	1.09	3.74	3.49	4.01	1.08	0.99	1.18	0.92	0.68	1.25	0.99	0.88	1.12
**Cervical cancer**															
** Cancer care utilization pattern**															
Within-region	1.00			1.00			1.00			1.00			1.00		
Out-of-region	1.03	0.99	1.06	1.16	0.98	1.36	1.20	0.96	1.51	1.25	0.78	2.01	0.96	0.77	1.20
** Treatment location and fragmented care**															
WR/NF	1.00			1.00			1.00			1.00			1.00		
OR/NF	1.02	0.98	1.06	1.13	0.95	1.35	1.18	0.93	1.5	1.4	0.84	2.31	0.92	0.73	1.17
WR/WF	0.99	0.94	1.05	3.06	2.46	3.8	1.15	0.83	1.59	1.39	0.75	2.58	0.85	0.62	1.17
OR/WF	1.06	1.01	1.1	3.77	3.08	4.62	1.48	1.13	1.96	1.15	0.92	2.15	0.96	0.73	1.26

RR, relative risk; OR, odds ratio; HR, hazard ratio; CI, confidence interval; WR/NF, within-region care without pretreatment care-seeking fragmentation; OR/NF, out-of-region care without pretreatment care-seeking fragmentation; WR/WF, within-region care with pretreatment care-seeking fragmentation; OR/WF, out-of-region care with pretreatment care-seeking fragmentation.

**Table A7:** Covariate balance after propensity score matching.

Variable	Cancer Care Utilization Pattern				
	Within-Region		Out-of-Region		Standardized Mean Difference
	N	%	N	%	
**Total**	102,180	50.0	102,180	50.0	
**Age**					
30s	4147	51.1	3972	48.9	−0.0095
40s	15,895	50.3	15,703	49.7	−0.0053
50s	26,897	50.8	26,001	49.2	−0.0202
60s and older	55,241	49.4	56,504	50.6	0.025
**Sex**					
Male	49,810	49.6	50,598	50.4	0.0154
Female	52,370	50.4	51,582	49.6	−0.0154
**Residential** **region**					
Metropolitan	43,762	50.7	42,505	49.3	−0.0247
Rural	58,418	49.5	59,675	50.5	0.0247
**Income**					
Q1 (lowest)	24,157	50.6	23,557	49.4	−0.0135
Q2	14,454	51.6	13,562	48.4	−0.0252
Q3	17,099	50.8	16,529	49.2	−0.015
Q4	20,627	49.8	20,800	50.2	0.0042
Q5 (highest)	25,843	48.2	27,732	51.8	0.0433
**Health insurance type**					
Medical Aid	5577	52.6	5021	47.4	−0.0218
NHI Self-employed	32,570	51.3	30,915	48.7	−0.0351
NHI Employee	64,033	49.2	66,244	50.8	0.0448
**Disability**					
Non-disabled	89,750	49.7	90,830	50.3	0.0328
Disabled	12,430	52.3	11,350	47.7	−0.0328
**CCI**					
0	72,002	49.2	74,316	50.8	−0.0476
1	12,487	52.1	11,486	47.9	
≥2	17,691	51.9	16,378	48.1	
**SEER stage**					
Localized	54,216	49.1	56,178	50.9	0.0387
Regional	38,334	50.6	37,459	49.4	−0.0179
Distant	6392	52.7	5741	47.3	−0.0282
Unknown	3238	53.6	2802	46.4	−0.0249
**Cancer treatment**					
Surgery	57,632	49.5	58,905	50.5	0.0252
Surgery with chemo or radiotherapy	44,548	50.7	43,275	49.3	−0.0252
**Types of cancer**					
Gastric	29,732	48.8	31,252	51.2	0.032
Colorectal	25,760	51.0	24,772	49.0	−0.0224
Liver	10,762	50.4	10,607	49.6	−0.0053
Lung	9530	49.6	9689	50.4	0.0058
Breast	23,653	50.1	23,590	49.9	−0.0014
Cervical	2743	54.7	2270	45.3	−0.0293
**Year of cancer diagnosis**					
2012	10,773	49.8	10,868	50.2	0.006
2013	11,295	50.6	11,030	49.4	
2014	11,219	50.3	11,076	49.7	
2015	11,267	50.2	11,156	49.8	
2016	12,078	50.3	11,940	49.7	
2017	11,782	49.7	11,922	50.3	
2018	11,706	49.1	12,116	50.9	
2019	11,067	49.4	11,323	50.6	
2020	10,993	50.6	10,749	49.4	

NHI, National Health Insurance; SEER, Surveillance, Epidemiology, and End Results; CCI, Charlson Comorbidity Index.
